# Effect of GH Deficiency Caused by Nonfunctioning Pituitary Masses on Serum C-reactive Protein Levels

**DOI:** 10.1210/jendso/bvad137

**Published:** 2023-11-04

**Authors:** Yasufumi Seki, Satoshi Morimoto, Kanako Bokuda, Daisuke Watanabe, Kaoru Yamashita, Noriyoshi Takano, Kosaku Amano, Takakazu Kawamata, Atsuhiro Ichihara

**Affiliations:** Department of Internal Medicine, Tokyo Women's Medical University, Tokyo, 162-8666, Japan; Department of Internal Medicine, Tokyo Women's Medical University, Tokyo, 162-8666, Japan; Department of Internal Medicine, Tokyo Women's Medical University, Tokyo, 162-8666, Japan; Department of Internal Medicine, Tokyo Women's Medical University, Tokyo, 162-8666, Japan; Department of Internal Medicine, Tokyo Women's Medical University, Tokyo, 162-8666, Japan; Department of Internal Medicine, Tokyo Women's Medical University, Tokyo, 162-8666, Japan; Department of Neurosurgery, Tokyo Women's Medical University, Tokyo, 162-8666, Japan; Department of Neurosurgery, Tokyo Women's Medical University, Tokyo, 162-8666, Japan; Department of Internal Medicine, Tokyo Women's Medical University, Tokyo, 162-8666, Japan

**Keywords:** hypopituitarism, inflammation, pituitary neuroendocrine tumor, Rathke's cleft cyst

## Abstract

**Context:**

GH supplementation for GH deficiency (GHD) has been reported to decrease high-sensitivity C-reactive protein (hs-CRP), an inflammatory marker; however, the association between GHD and hs-CRP remains unclear.

**Objective:**

We aimed to clarify the impact of impaired GH secretion due to pituitary masses on hs-CRP levels.

**Methods:**

We retrospectively examined the association between GH secretion, assessed using GH-releasing peptide-2, and serum hs-CRP levels before and a year after the pituitary surgery in patients with nonfunctioning pituitary neuroendocrine tumor or Rathke cleft cyst.

**Results:**

Among 171 patients, 55 (32%) presented with severe GHD (peak GH response to GH-releasing peptide-2 < 9 ng/mL). Serum hs-CRP levels were significantly higher in patients with severe GHD than in those without (*P* < .001) and significantly correlated with the peak GH (*r* = −0.50, *P* < .001). Multiple regression analyses showed that the peak GH significantly and negatively predicted hs-CRP levels (β = −0.345; 95% CI, −0.533 to −0.158) and the lowest quartile of the peak GH (<5.04 ng/mL) were significantly associated with increase in hs-CRP levels (exp [β] = 1.840; 95% CI, 1.209 to 2.801), after controlling for other anterior hormones and metabolic parameters. Postoperative change in the peak GH (N = 60) significantly predicted change in hs-CRP levels (β = −0.391; 95% CI, −0.675 to −0.108), independent of alterations in other anterior hormones and metabolic parameters.

**Conclusion:**

The inverse association between GH secretion and hs-CRP levels highlights the protective role of GH in the increase in hs-CRP.

GH deficiency (GHD) is the most common endocrine deficit complicated by pituitary tumors [1] and is known to cause metabolic complications, such as obesity, dyslipidemia [2], and fatty liver [3], and premature atherosclerosis [4]. The major causes of adult-onset GHD are nonfunctioning pituitary neuroendocrine tumors (NF-PitNETs) and Rathke cleft cysts (RCCs) [5]. Patients with GHD reportedly present higher mortality and cardiovascular event risks than age- and sex-matched controls [6, 7]. Furthermore, an observational study reported a decreased risk of cardiovascular events after GH supplementation therapy [7]. Therefore, GHD is believed to play a role in the pathogenesis of atherosclerosis, leading to cardiovascular events.

Atherosclerosis is characterized by a low-grade chronic inflammation of the arterial wall [8]. C-reactive protein (CRP) is an established inflammatory biomarker and 1 of the most widely used biomarkers associated with the risk of cardiovascular events [9, 10]. Patients with hypopituitarism have been observed to exhibit elevated CRP levels [11]. Serum high-sensitivity CRP (hs-CRP) levels decrease after GH supplementation in GH-deficient patients [12, 13]. These reports suggest an important role for GHD in the development of inflammation in patients with hypopituitarism; however, the association between impaired GH secretion and the development of inflammation remains unclear.

In the present study, we aimed to clarify the role of impaired GH secretion in the increase in CRP levels. We retrospectively investigated the association between GH secretion and serum hs-CRP levels in patients with NF-PitNET and RCC.

## Materials and Methods

### Study Population

In this retrospective cross-sectional study, we reviewed all available charts of adult patients with NF-PitNET or RCC who were admitted to the Department of Endocrinology and Hypertension at Tokyo Women's Medical University Hospital between January 2014 and March 2018. Patients with NF-PitNET or RCC who underwent GH-releasing peptide (GHRP)-2 testing and serum hs-CRP measurements were included in this study. Patients with an estimated glomerular filtration rate (GFR) of <30 mL/min/1.73 m^2^, active inflammatory or malignant diseases, or a history of pituitary surgery, or under GH supplementation therapy were excluded. Background clinical characteristics including age, sex, body mass index (BMI), comorbidities, renal function, pituitary function, and pituitary mass size were retrieved. In patients who underwent pituitary surgery, serum hs-CRP levels, peak GH response to GHRP-2, and metabolic parameters a year after pituitary surgery were also retrieved. Obesity was defined as a BMI ≥25 kg/m^2^. This study was conducted in accordance with the 1964 Declaration of Helsinki and its amendments. Because the study was defined as one without human samples under the Japanese guidelines presented by the Ministry of Health, Labour and Welfare, written informed consent was not required, and we used our official institutional website as an opt-out method. This study was approved by the Ethics Committee of the Tokyo Women's Medical University Hospital (4856-R) and was designed and conducted in accordance with Strengthening the Reporting of Observational Studies in Epidemiology guidelines [14].

### GHRP-2 Test

All patients with nonfunctioning pituitary masses underwent GHRP-2 tests to determine GH secretion. Briefly, pralmorelin hydrochloride (0.1 mg) (Kaken Pharmaceutical Co. Ltd., Tokyo, Japan) was IV injected at 9 Am, and blood samples were collected before and 15, 30, and 45 minutes after injection. Severe GHD was defined as a peak GH concentration <9 ng/mL [15]. A GH cutoff value of 9 ng/mL with GHRP-2 corresponded to a GH value of 1.8 ng/mL with an insulin tolerance test when the GH value was calibrated with the recombinant World Health Organization 98/574 standard [15]. Although the GHRP-2 test is not included in the Endocrine Society Guidelines [16], it is considered safe [17] and is widely used to diagnose severe GHD in Japan because of its high sensitivity and specificity compared with those of an insulin tolerance test [18].

### Assays and Measurements

Serum aspartate aminotransferase (AST) (Quick Auto Neo AST; Shino-Test, Tokyo, Japan), alanine aminotransferase (ALT) (Quick Auto Neo ALT; Shino-Test), γ-glutamyl transpeptidase (GGT) (Quick Auto Neo γ-GT; Shino-Test), and creatinine (Cygnus Auto Cre; Shino-Test) were measured using the enzymatic methods. Hemoglobin A1c (HbA1c) was measured using HPLC (HA-8190 V, Arkley, Kyoto, Japan) per the National Glycohemoglobin Standardization Program. Serum high-density lipoprotein (HDL)-cholesterol (MetaboLead HDL-C; Minaris Medical, Tokyo, Japan), low-density lipoprotein (LDL)-cholesterol (MetaboLead LDL-C; Minaris Medical), and triglyceride (Deteminer L TG II; Minaris Medical) levels were measured using colorimetric enzymatic methods. Elevated liver enzyme levels were defined as serum AST, ALT, or GGT levels higher than the upper limit of normal. The estimated GFR was calculated using a formula developed by the Japanese Society of Nephrology [19]. Serum hs-CRP concentrations were measured using a latex nephelometry assay, N-latex CRP-2 (Siemens Diagnostics Japan, Tokyo, Japan). A high hs-CRP level was defined as a serum hs-CRP level higher than 1000 ng/mL. Serum GH, free thyroxine, and total testosterone concentrations were measured using an electrochemiluminescence immunoassay, Elecsys hGH (Roche, RRID: AB_2883977), Elecsys FT4 II (Roche, RRID: AB_2924686), and Elecsys testosterone II (Roche, RRID: AB_2783736), respectively. In addition, serum IGF-1 concentrations were measured using an immunoradiometric assay, IGF-1 (Somatomedin-C) IRMA (Daiichi) (Fujirebio, RRID: AB_3073548). The IGF-1 SD score was calculated based on age- and sex-specific normative IGF-1 data from the Japanese population [20]. We calculated the pituitary mass volume using the formula for an ellipsoid approximation, π/6 × (A × B × C), where A, B, and C are the anteroposterior, vertical, and transverse diameters of the masses, respectively [21].

### Statistical Analyses

Statistical analyses were performed using JMP Pro software (version 16.0; SAS Institute, USA). Data are presented as mean ± SD or median (interquartile range) for descriptive analyses. Data normality was analyzed by visual inspection of the Q–Q plots and histograms. Given the skewed distribution, the peak GH response to GHRP-2, serum levels of hs-CRP, AST, ALT, GGT, HDL-cholesterol, LDL-cholesterol, and triglyceride, and pituitary mass volume were log-transformed for the analyses that follow. Statistical significance between 2 groups was calculated using the unpaired Student *t* test. Statistical significance between the values before and a year after pituitary surgery was calculated using the paired Student *t* test. Multiple comparisons among the groups were performed using the Tukey test. The significance of categorical data was assessed using Fisher exact test. Univariate regression analyses were performed using Pearson correlation coefficients. Analysis of covariance was used to determine the differences between NF-PitNET and RCC after adjusting for mass size. Because background characteristics, including other pituitary hormone secretions may bias the association between GH secretion and hs-CRP levels in multiple regression analyses, to estimate serum hs-CRP levels, explanatory factors were selected from peak GH response to GHRP-2; elevated liver enzyme levels as a complication of GHD; other anterior pituitary hormone deficiencies; and known factors associated with serum hs-CRP levels such as conventional coronary heart disease risk factors including age, sex, BMI, hypertension, diabetes mellitus, and hyperlipidemia [22], and serum creatinine level [23]. In the analyses, peak GH response to GHRP-2 as an explanatory variable was used as a continuous variable or as a quartile group and thyroid function as a tertile group. The standardized β coefficient was calculated to compare the strength of each independent variable. We conducted a sensitivity analysis for patients without corticotroph deficiency, hypothyroidism, or hypogonadotropic hypogonadism. The number of cases during the study period determined the sample size. No missing data were imputed. Statistical significance was set at *P* < .05.

## Results

### Characteristics of Study Participants

**Figure 1.**
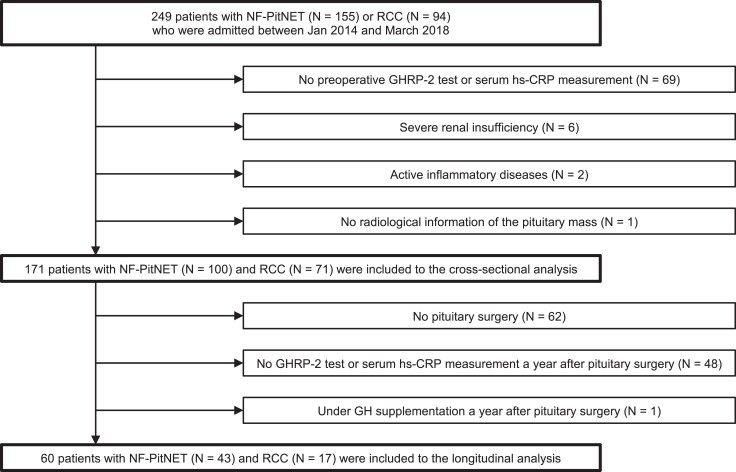
Flowchart of study population enrollment. Abbreviations: GHRP, GH-releasing peptide; hs-CRP, high-sensitivity C-reactive protein; NF-PitNET, nonfunctioning pituitary neuroendocrine tumor; RCC, Rathke cleft cyst.

Adult patients with NF-PitNET (N = 155) and RCC (N = 94) were identified; those without preoperative GHRP-2 test or serum hs-CRP measurement (N = 69) and radiological information (N = 1) and those with severe renal insufficiency (N = 6) and inflammatory diseases (N = 2) were excluded (Fig. 1). Thus, 171 patients with NF-PitNET (N = 100) or RCC (N = 71) were included in our study.

**Figure 2.**
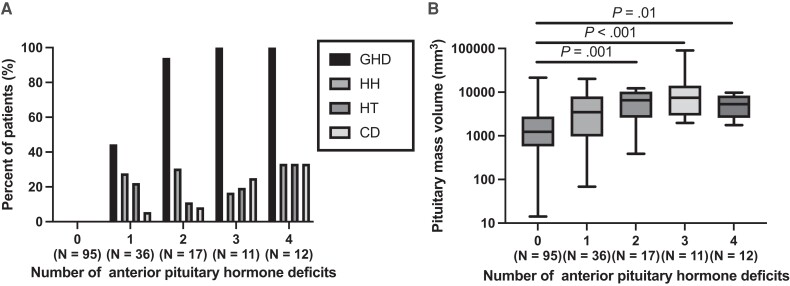
Characteristics of the pituitary function of the patients. (A) Association between the prevalence of GHD, hypogonadotropic hypogonadism, hypothyroidism, and corticotroph deficiency, and the deficit number of anterior pituitary hormones. (B) Association between the pituitary mass volume and the number of anterior pituitary hormone deficits. Abbreviations: CD, corticotroph deficiency; GHD, GH deficiency; HH, hypogonadotropic hypogonadism; HT, hypothyroidism; NF-PitNET, nonfunctioning pituitary neuroendocrine tumor; RCC, Rathke cleft cyst.

Patient background characteristics are listed in Table 1. Of the 171 patients, 95 (56%) had no anterior pituitary deficiencies, 26 (15%) had corticotroph deficiency, 31 (18%) had hypothyroidism, 39 (23%) had hypogonadotropic hypogonadism, and 55 (32%) had severe GHD. Severe isolated GHD was observed in 16 patients (9%). Most patients with 2 or more anterior pituitary hormone deficiencies had severe GHD (Fig. 2A). Overall, 15 (9%) patients received hydrocortisone and 9 (5%) received levothyroxine. The clinical diagnosis of the pituitary mass was pathologically confirmed in 109 (64%) of the 171 patients.

**Table 1.** Background characteristics

	N	Overall (N = 171)	Severe GHD− (N = 116)	Severe GHD+ (N = 55)	*P*
Age, y	171	54 ± 16	52 ± 17	56 ± 14	.13
Male sex, N (%)	171	67 (39)	37 (32)	30 (55)	.007
BMI, kg/m^2^	171	23.5 ± 3.8	23.0 ± 3.7	24.6 ± 3.7	.009
BMI >25 kg/m^2^		53 (31)	28 (24)	25 (35)	.008
Hypertension, N (%)	171	57 (33)	36 (31)	21 (38)	.39
Diabetes mellitus, N (%)	171	25 (15)	15 (13)	10 (18)	.36
Dyslipidemia, N (%)	171	115 (67)	71 (61)	44 (80)	.02
Clinical diagnosis, N (%)	171				
NF-PitNET		100 (58)	61 (53)	39 (71)	.02
RCC		71 (42)	55 (47)	16 (29)	
Pituitary mass volume, mm^3^	171	2198 (777-5526)	1378 (554-3235)	6416 (2753-9734)	<.001
Number of anterior pituitary hormone deficits, N (%)	171				
0		95 (56)	95 (82)	0 (0)	<.001
1		36 (21)	20 (17)	16 (29)	
2		17 (10)	1 (1)	16 (29)	
3		11 (6)	0 (0)	11 (20)	
4		12 (7)	0 (0)	12 (22)	
Corticotroph deficiency, N (%)	171	26 (15)	2 (2)	24 (44)	<.001
Hypothyroidism, N (%)	171	31 (18)	9 (8)	22 (40)	<.001
Hypogonadotropic hypogonadism, N (%)	171	39 (23)	11 (9)	28 (51)	<.001
hs-CRP, ng/mL	171	348 (148-796)	249 (113-537)	754 (393-1330)	<.001
AST, U/L	171	20 (17-26)	20 (15-25)	23 (19-31)	.001
ALT, U/L	171	17 (12-25)	16 (12-23)	19 (14-28)	.03
GGT, U/L	170	20 (14-33)	18 (13-31)	24 (17-42)	.01
Elevated liver enzymes, N (%)	171	42 (25)	22 (19)	20 (36)	.02
HbA1c, %	171	5.7 (5.5-6.1)	5.7 (5.5-6.0)	5.8 (5.4-6.1)	.30
Creatinine, mg/dL	171	0.70 (0.61-0.83)	0.68 (0.59-0.78)	0.79 (0.63-0.86)	.003
Estimated GFR, mL/min/1.73 m^2^	171	77.6 ± 15.7	79.2 ± 15.7	74.1 ± 15.4	.05
HDL-cholesterol, mg/dL	171	56 (48-70)	61 (50-73)	53 (41-59)	.001
LDL-cholesterol, mg/dL	171	121 (100-146)	112 (93-134)	136 (115-162)	<.001
Triglyceride, mg/dL	171	96 (71-132)	86 (64-119)	121 (90-181)	<.001
Peak GH response to GHRP-2, ng/mL	171	16.1 (5.0-34.8)	27.0 (15.6-43.6)	2.5 (1.3-4.7)	<.001
IGF-1, ng/mL	171	104 (77-141)	122 (91-149)	75 (51-106)	<.001
IGF-1 SD score	171	−1.1 ± 1.4	−0.7 ± 1.2	−2.0 ± 1.5	<.001
Free thyroxine, ng/mL	171	1.14 ± 0.26	1.23 ± 0.21	0.96 ± 0.26	<.001
Total testosterone (male), ng/mL	67	357 ± 213	475 ± 146	212 ± 193	<.001

Data are presented as mean ± SD or median (interquartile range).

Abbreviations: ALT, alanine aminotransferase; AST, aspartate aminotransferase; BMI, body mass index; GGT, γ-glutamyl transpeptidase; GFR, glomerular filtration rate; GHD, GH deficiency; GHRP-2, GH-releasing peptide-2; HbA1c, hemoglobin A1c; HDL, high-density lipoprotein; hs-CRP, high-sensitivity C-reactive protein; LDL, low-density lipoprotein; NF-PitNET, nonfunctioning pituitary neuroendocrine tumor; RCC, Rathke cleft cyst.

Mass volumes in patients with NF-PitNETs were significantly higher than those in patients with RCCs (4596 [1925–8949] vs 1093 [487–2029] mm^3^, *P* < .001). The mass volumes in patients with 2 (*P* = .001), 3 (*P* < .001), or 4 (*P* = .01) deficits in the anterior pituitary hormones were significantly higher than those in patients without anterior pituitary hormone deficiencies (Fig. 2B).

### Association Between Serum hs-CRP Level and Background Characteristics

The serum hs-CRP level was 348 (148-796) ng/mL, and high hs-CRP levels were observed in 35 (20%) patients. Male sex (*P* < .001), obesity (*P* < .001), and dyslipidemia (*P* = .02) were significantly associated with increased serum hs-CRP levels. Hypertension (*P* = .37) and diabetes mellitus (*P* = .05) were not significantly associated with serum hs-CRP levels. Serum hs-CRP levels were significantly positively correlated with age, BMI, and levels of AST, ALT, GGT, creatinine, LDL-cholesterol, triglyceride, and HbA1c, and negatively correlated with estimated GFR, and HDL-cholesterol levels (Table 2).

**Table 2.** Correlation between serum hs-CRP levels*^a^* and background characteristics

Variables	*r*	*P*
Age	0.19	.01
BMI	0.42	<.001
AST*^a^*	0.32	<.001
ALT*^a^*	0.37	<.001
GGT*^a^*	0.38	<.001
Creatinine	0.28	<.001
Estimated GFR	−0.22	.004
HbA1c	0.18	.02
HDL-cholesterol*^a^*	−0.23	.002
LDL-cholesterol*^a^*	0.19	.01
Triglyceride*^a^*	0.31	<.001

Abbreviations: ALT, alanine aminotransferase; AST, aspartate aminotransferase; BMI, body mass index; GGT, γ-glutamyl transpeptidase; GFR, glomerular filtration rate; HbA1c, hemoglobin A1c; HDL, high-density lipoprotein; hs-CRP, high-sensitivity C-reactive protein; LDL, low-density lipoprotein.

^*a*^Values were log-transformed for analyses.

### Association Between Serum hs-CRP Level and the Characteristics of Pituitary Masses

**Figure 3.**
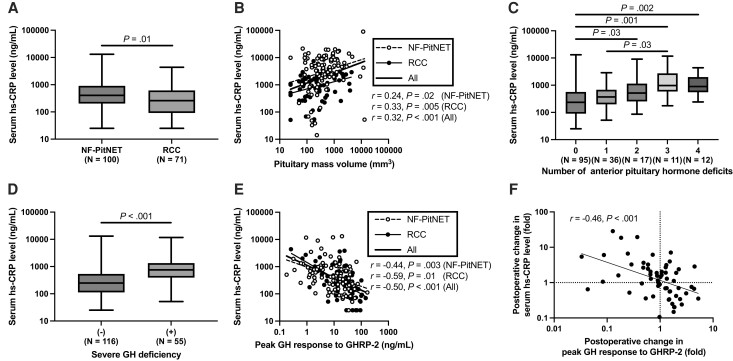
Association between serum hs-CRP levels and background characteristics. (A) Serum hs-CRP levels in the patients with NF-PitNET and RCC. (B) Correlation between serum hs-CRP levels and pituitary mass volumes in patients with NF-PitNET (open circles and dotted line) and RCC (closed circles and solid line). The bold solid line represents the regression slope in all the patients. (C) Association between serum hs-CRP levels and the number of anterior pituitary hormone deficits. (D) Serum hs-CRP levels in patients with and without severe GHD. (E) Correlation between serum hs-CRP levels and peak GH response to GHRP-2 in patients with NF-PitNET (open circles and dotted line) and RCC (closed circles and solid line). The bold solid line represents the regression slope in all the patients. (F) Correlation between changes in serum hs-CRP levels and peak GH response to GHRP-2 before and a year after pituitary surgery. The solid line represents the regression slope. Abbreviations: GHD, GH deficiency; GHRP, GH-releasing peptide; hs-CRP, high-sensitivity C-reactive protein; NF-PitNET, nonfunctioning pituitary neuroendocrine tumor; RCC, Rathke cleft cyst.

We assessed the association between serum hs-CRP levels and the pituitary mass characteristics. Serum hs-CRP levels were significantly higher in patients with NF-PitNET than in those with RCC (405 [203-907] vs 260 [92-616] ng/mL, *P* = .02) (Fig. 3A). Serum hs-CRP levels were significantly correlated with mass volume in patients with NF-PitNET (*r* = 0.24, *P* = .02) and RCC (*r* = 0.33, *P* = .005), and in all patients (*r* = 0.32, *P* < .001) (Fig. 3-B). Analysis of covariance revealed that mass volume (β = 0.354, *P* < .001), but not RCC (β = −0.047, *P* = .58) or the interaction between mass volume and RCC (β = 0.125, *P* = .15) was a significant explanatory variable for estimating serum hs-CRP levels.

### Association Between Serum hs-CRP Level and Anterior Pituitary Hormones

Serum hs-CRP levels in patients with 2 (*P* = .03), 3 (*P* = .001), or 4 (*P* = .002) deficits of anterior pituitary hormones were significantly higher than those in patients without anterior pituitary hormone deficiency (Fig. 3C). Serum hs-CRP levels were significantly higher in patients with severe GHD than in those without severe GHD (754 [393-1330] vs 249 [113-537] ng/mL, *P* < .001) (Fig. 3D) and were significantly correlated with the peak GH response to GHRP-2 (*r* = −0.50, *P* < .001) (Fig. 3E) but not with the IGF-1 SD score (*r* = −0.10, *P* = .21). The correlation between serum hs-CRP levels and peak GH response to GHRP-2 was significant in patients with NF-PitNET (*r* = −0.44, *P* = .003) and RCC (*r* = −0.59, *P* = .01). Serum hs-CRP levels were also significantly and inversely correlated with free thyroxine levels (*r* = −0.22, *P* = .003) and were significantly higher in patients with hypothyroidism than in those without hypothyroidism (610 [235-1270] vs 302 [125-711] ng/mL, *P* = .04). Serum hs-CRP levels in patients with hypogonadotropic hypogonadism (586 [348-1240] vs 272 [124-688] ng/mL, *P* < .001) and corticotroph deficiency (997 [577-2058] vs 283 [125-610] ng/mL, *P* < .001) were significantly higher than those in patients without hypogonadotropic hypogonadism and corticotroph deficiency, respectively.

### Multiple Linear Regression Analyses With Serum hs-CRP Level

In the multiple linear regression analysis (Table 3), the peak GH response to GHRP-2 (β = −0.376; 95% CI, −0.511 to −0.240; standardized β = −0.413) was a significant variable for determining serum hs-CRP levels after adjusting for age, sex, hypertension, diabetes mellitus, dyslipidemia, and serum creatinine level (model 1). When corticotroph deficiency, hypogonadotropic hypogonadism, and thyroid function were added to the model (model 2), the peak GH response to GHRP-2 (β = −0.342; 95% CI, −0.537 to −0.148; standardized β = −0.377) was also a significant variable. When elevated liver enzyme levels were further included in the model (model 3), the peak GH response to GHRP-2 (β = −0.345; 95% CI, −0.533 to −0.158; standardized β = −0.380) was a significant variable. The standardized β coefficients of the peak GH response to GHRP-2 were larger than those of the other variables in model 2 and model 3. When the patients with anterior pituitary hormone deficiencies other than GH were excluded (N = 111), peak GH response to GHRP-2 was also a significant variable for determining serum hs-CRP levels in model 1 (β = −0.446; 95% CI, −0.701 to −0.191; standardized β = −0.315).

**Table 3.** Multiple linear regression analyses with the serum hs-CRP levels*^a^*

Variables	Model 1		Model 2		Model 3	
	β (95% CI)	Standardized β	β (95% CI)	Standardized β	β (95% CI)	Standardized β
Overall (N = 171)						
Peak GH response to GHRP-2*^a^*, ng/mL	−0.376 (−0.511 to −0.240)	−0.413	−0.342 (−0.537 to −0.148)	−0.377	−0.345 (−0.533 to −0.158)	−0.380
Corticotroph deficiency	—		0.077 (−0.241 to 0.396)	0.044	0.138 (−0.172 to 0.447)	0.078
Thyroid function						
T1 (free T4 < 1.05 ng/dL)	—		reference		reference	
T2 (free T4 ≥ 1.05, <1.25 ng/dL)	—		−0.045 (−0.287 to 0.198)	−0.029	−0.043 (−0.277 to 0.191)	−0.028
T3 (free T4 > 1.25 ng/dL)	—		0.070 (−0.186 to 0.326)	0.045	0.096 (−0.151 to 0.334)	0.062
Hypogonadotropic hypogonadism	—		0.058 (−0.183 to 0.299)	0.038	−0.024 (−0.260 to 0.213)	−0.016
Elevated liver enzymes	—		—		0.379 (0.167 to 0.592)	0.258
Adjusted *R*^2^ and *P* value for the whole model	*R* ^2^ = 0.277 *P* < .001		*R* ^2^ = 0.263 *P* < .001		*R* ^2^ = 0.313 *P* < .001	
Patients without corticotroph deficiency, hypothyroidism, or hypogonadotropic hypogonadism (N = 111)						
Peak GH response to GHRP-2*^a^*, ng/mL	−0.446 (−0.701 to −0.191)	−0.338	—		—	
Adjusted *R*^2^ and *P* value for the whole model	*R* ^2^ = 0.183 *P* < .001					

Model 1: adjusted for age, sex, obesity, hypertension, diabetes mellitus, dyslipidemia, and creatinine level. Model 2: adjusted for factors used in model 1 + corticotroph deficiency, thyroid function, and hypogonadotropic hypogonadism. Model 3: adjusted for the factors used in model 2 + elevated liver enzymes.

Abbreviations: GHRP, GH-releasing peptide; hs-CRP, high-sensitivity C-reactive protein.

^*a*^Values were log-transformed for analyses.

**Figure 4.**
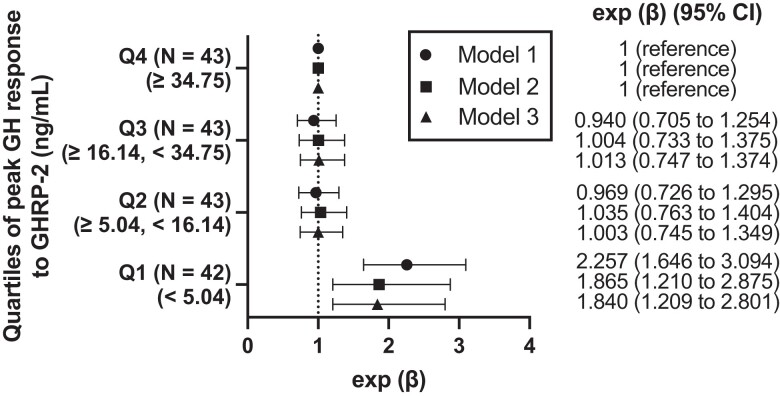
Association between serum hs-CRP levels and GH secretion. Forest plot demonstrating exponential transformation of β coefficients (exp [β]) and 95% CIs of quartiles of peak GH response to GHRP-2 levels in the multivariable regression analyses to estimate serum hs-CRP levels, with the highest quartile (Q4) as reference. Because the dependent variable was log-transformed, the β coefficients were exponentially transformed. Exponential transformation of β coefficients (Exp [β]) implies an interpretation on a multiplicative scale. Exp (β) implies an interpretation on a multiplicative scale. The serum hs-CRP level changes by 100 × (exp [β])% in the quartiles of peak GH response to GHRP-2 levels compared with Q4. Model 1: adjusted for age, sex, obesity, hypertension, diabetes mellitus, dyslipidemia, and creatinine level. Model 2: adjusted for factors used in model 1 + corticotroph deficiency, thyroid function, and hypogonadotropic hypogonadism. Model 3: Adjusted for the factors used in model 2 + elevated liver enzymes. Abbreviations: GHRP, GH-releasing peptide; hs-CRP, high-sensitivity C-reactive protein.

The lowest quartile group (Q1) (peak GH response to GHRP-2 < 5.04 ng/mL) (exp [β] = 2.257; 95% CI, 1.646 to 3.094) was significantly associated with an increase in serum hs-CRP levels compared with the highest quartile group (Q4) (peak GH response to GHRP-2 ≥ 34.75 ng/mL) after adjusting for age, sex, obesity, hypertension, diabetes mellitus, dyslipidemia, and serum creatinine level (model 1) (Fig. 4). When corticotroph deficiency, thyroid function, and hypogonadotropic hypogonadism were included in the model (model 2), Q1 was also significantly associated with the serum hs-CRP levels (exp [β] = 1.865; 95% CI, 1.210 to 2.875). When elevated liver enzyme level was further included in the model (model 3), Q1 was also significantly associated with the serum hs-CRP levels (exp [β] = 1.840; 95% CI, 1.209 to 2.801).

### Postoperative Changes in Serum hs-CRP Level and GH Secretion

Of the 108 patients who underwent pituitary surgery (75 with NF-PitNET and 33 with RCC), 61 underwent postoperative serum hs-CRP measurements and GHRP-2 tests a year after pituitary surgery. After excluding 1 patient who was receiving GH supplementation therapy, the remaining 60 patients (43 with NF-PitNET and 17 with RCC) were included in the longitudinal analyses (Fig. 1). Table 4 shows the postoperative changes in clinical characteristics.

**Table 4.** Postoperative changes in pituitary function and metabolic parameters in the 60 patients with postoperative assessments

	Baseline	A year after pituitary surgery	*P*
hs-CRP*^a^*, ng/mL	503 (242-1168)	609 (277-1460)	.15
BMI, kg/m^2^	23.8 ± 3.7	24.2 ± 3.8	.05
Systolic blood pressure, mm Hg	120 ± 16	119 ± 16	.41
AST*^a^*, U/L	21 (19-27)	22 (17-29)	.42
ALT*^a^*, U/L	18 (13-23)	18 (13-27)	.70
GGT*^a^*, U/L	21 (13-39)	21 (14-35)	.97
HbA1c, %	5.8 (5.5-6.0)	5.8 (5.4-6.1)	.64
HDL-cholesterol*^a^*, mg/dL	55 (46-62)	56 (44-66)	.96
LDL-cholesterol*^a^*, mg/dL	123 (106-143)	117 (101-132)	.26
Triglyceride*^a^*, mg/dL	99 (82-146)	115 (76-161)	.21
Peak GH response to GHRP-2*^a^*, ng/mL	8.9 (2.4-14.2)	7.3 (2.1-16.3)	.28
Severe GHD, N (%)	30 (50)	33 (55)	.37
Corticotroph deficiency, N (%)	20 (33)	23 (38)	.44
Free thyroxine, ng/mL	1.01 ± 0.25	1.12 ± 0.19	.001
Hypothyroidism, N (%)	20 (33)	23 (38)	.44
Hypogonadotropic hypogonadism, N (%)	21 (35)	19 (32)	.62

Data are presented as mean ± SD or median (interquartile range).

Abbreviations: ALT, alanine aminotransferase; AST, aspartate aminotransferase; BMI, body mass index; GGT, γ-glutamyl transpeptidase; GHD, GH deficiency; GHRP, GH-releasing peptide; HbA1c, hemoglobin A1c; HDL, high-density lipoprotein; hs-CRP, high-sensitivity C-reactive protein; LDL, low-density lipoprotein.

^*a*^Values were log-transformed for analyses.

Postoperative changes in serum hs-CRP levels were significantly and inversely correlated with those in peak GH response to GHRP-2 (*r* = −0.46, *P* < .001) (Fig. 3F). In addition, the postoperative changes in serum hs-CRP levels were also significantly correlated with those in serum HDL-cholesterol levels (*r* = −0.40, *P* = .002) but not in BMI, systolic blood pressure, or serum levels of AST, ALT, GGT, HbA1c, LDL-cholesterol, and triglyceride (Table 5). Multiple linear regression analyses revealed that the postoperative change in the peak GH response to GHRP-2 (β = −0.392; 95% CI, −0.673 to −0.111; standardized β = −0.354) was a significant factor in determining the change in serum hs-CRP levels after adjusting for changes in anterior pituitary hormone secretion other than GH (Table 6). When the model was further extended to include BMI, systolic blood pressure, and serum levels of ALT and HDL-cholesterol, the change in the peak GH response to GHRP-2 (β = −0.391; 95% CI, −0.675 to −0.108; standardized β = −0.353) was also a significant factor in determining the change in serum hs-CRP levels.

**Table 5.** Correlation between the postoperative changes in serum hs-CRP levels*^a^* and those in metabolic parameters characteristics

Variables	*r*	*P*
BMI	0.07	.58
Systolic blood pressure	0.09	.47
AST*^a^*	0.12	.35
ALT*^a^*	−0.02	.89
GGT*^a^*	−0.13	.34
HbA1c	0.11	.40
HDL-cholesterol*^a^*	−0.40	.002
LDL-cholesterol*^a^*	0.19	.15
Triglyceride*^a^*	0.13	.34

Abbreviations: ALT, alanine aminotransferase; AST, aspartate aminotransferase; BMI, body mass index; GGT, γ-glutamyl transpeptidase; HbA1c, hemoglobin A1c; HDL, high-density lipoprotein; hs-CRP, high-sensitivity C-reactive protein; LDL, low-density lipoprotein.

^*a*^Values were log-transformed for analyses.

**Table 6.** Multiple linear regression analyses with the postoperative changes in serum hs-CRP levels*^a^*

Variables	Model 1		Model 2	
	β (95% CI)	Standardized β	β (95% CI)	Standardized β
Postoperative changes in				
Peak GH response to GHRP-2*^a^*, ng/mL	−0.392 (−0.673 to −0.111)	−0.354	−0.391 (−0.675 to −0.108)	−0.353
Number of anterior hormone deficiencies (excluding GH)	0.110 (−0.004 to 0.223)	0.245	0.089 (−0.027 to 0.205)	0.199
BMI, kg/m^2^	—		−0.024 (−0.110 to 0.063)	−0.072
SBP, mm Hg	—		0.003 (−0.005 to 0.010)	0.083
ALT*^a^*, U/L	—		0.079 (−0.532 to 0.689)	0.032
HDL-cholesterol*^a^*, mg/dL	—		−1.931 (−3.362 to −0.499)	−0.317
Adjusted *R*^2^ and *P* value for the whole model	*R* ^2^ = 0.236 *P* < .001		*R* ^2^ = 0.300 *P* < .001	

Model 1: the model includes postoperative changes in peak GH response to GHRP-2 and number of anterior hormone deficiencies (excluding GH) as explanatory factors. Model 2: adjusted for factors used in model 1 + postoperative changes in BMI, SBP, ALT, and HDL-cholesterol.

Abbreviations: ALT, alanine aminotransferase; BMI, body mass index; GHRP, GH-releasing peptide; HDL, high-density lipoprotein; hs-CRP, high-sensitivity C-reactive protein; SBP, systolic blood pressure.

^*a*^Values were log-transformed for analyses.

## Discussion

In this retrospective study, serum hs-CRP levels were significantly correlated with the peak GH response to GHRP-2, as a marker of GH secretion, in patients with NF-PitNET and RCC. Although other pituitary hormone deficiencies including corticotroph deficiency, hypothyroidism, and hypogonadotropic hypogonadism were significantly associated with increased serum hs-CRP levels, the peak GH response to GHRP-2 was a significant variable for estimating serum hs-CRP levels after adjusting for other pituitary hormone secretions as well as atherosclerotic risk factors, and elevated liver enzymes as possible confounders of serum hs-CRP levels in multiple linear regression analyses. The lowest quartile group of the GH secretion was significantly associated with serum hs-CRP levels after the adjustment. Moreover, alterations in the peak GH response to GHRP-2 after pituitary surgery were significantly and inversely correlated with changes in serum hs-CRP levels, independent of changes in other anterior pituitary hormone secretions and metabolic parameters. These findings revealed a significant and inverse relationship between GH secretion and serum hs-CRP levels, independent of atherosclerotic risk factors, metabolic consequences of GHD including obesity and fatty liver, and other anterior pituitary hormone deficiencies.

There are 2 important findings regarding the association between impaired GH secretion and the increase in the inflammatory marker. First, impaired GH secretion may be a key factor associated with the increase in hs-CRP levels in patients with nonfunctioning pituitary mass lesions. Previous studies have established that patients with hypopituitarism exhibit increased CRP levels [11]. However, it was not clear which anterior pituitary hormone affected CRP levels. Our data show a significant association between the number of anterior pituitary hormone deficiencies and increased hs-CRP levels. Although adrenal [24], thyroid [25], and gonadal [26, 27] insufficiencies have been reportedly associated with increased inflammation, the results of this study suggest that GH secretion, as indicated by the peak GH response to GHRP-2, is an independent factor affecting serum hs-CRP levels, independent of other anterior hormone secretions. A previous in vivo study reported that IGF-1 administration decreased vascular inflammatory cytokines [28]. GHD, which leads to low IGF-1 levels, may also contribute to increase in inflammatory cytokines. Thus, impaired GH secretion resulting from nonfunctioning pituitary masses may play a pivotal role in the increase in CRP independent of other anterior pituitary hormones.

Second, GH-deficient states may be associated with an increase in the inflammatory marker, independent of its complications. GHD can cause obesity [29] and fatty liver [3]. A previous report demonstrated that white adipose tissue inflammation may contribute to elevated CRP levels in obese patients [30]. In addition, hepatic steatosis and obesity are independently associated with increased odds of high hs-CRP levels [31]. In the present study, high serum hs-CRP levels were associated with GHD independent of obesity and elevated liver enzyme levels. Similarly, serum CRP levels were decreased by GH supplementation therapy, independent of changes in body composition [12, 29]. Because the monocyte/macrophage–specific IGF1 receptor knockout model showed increased pro-inflammatory cytokine production in macrophages [32], a decrease in IGF-1 receptor signaling by GHD might lead to inflammation in macrophages and increase serum hs-CRP levels, at least in part, independent of the development of obesity and fatty liver. The lack of a significant correlation between IGF-1 SD scores and serum hs-CRP levels in our study may be explained by the fact that the main source of circulating IGF-1 is the liver [33] and its low diagnostic accuracy in estimating GH secretion [4]. Thus, impaired GH secretion may contribute to the increase in the inflammatory marker, independent of the metabolic complications of GHD. To the best of our knowledge, this is the first study to reveal a direct relationship between GH secretion and CRP levels independent of other pituitary hormone secretions and the complications of GHD.

Our findings indicate that assessing GH secretion in patients with pituitary disease is important for understanding the risk of cardiovascular events. Although observational studies have shown that patients receiving GH replacement have reduced mortality compared with patients without GH replacement, there is no convincing evidence that treatment of GHD improves mortality rates [34]. Glucocorticoid overreplacement and radiation therapy may bias the mortality of patients with GHD [34]. Consequently, adult GHD is perceived as having a low priority in some countries [35]. The direct association between impaired GH secretion and the inflammatory marker demonstrated in our study suggests an important protective role for GH in the development of cardiovascular diseases through inflammation.

Our study has some limitations. First, the mechanism linking the decrease in GH secretion to CRP production was not investigated. Pro-inflammatory cytokines such as IL-1 and IL-6 induce *CRP* gene expression [36]; however, the effect of GH supplementation on these cytokines has been contradictory [37]. Further studies on the association between GH secretion and cytokine levels are warranted. Second, the diagnosis of GHD was confirmed using the GHRP-2 test, which has only been approved in Japan [38]. However, a high correlation between the GH response to a GHRP-2 test and that to an insulin tolerance test has been reported [18]. The nonpeptide GH secretagogue macimorelin, approved in the United States and Europe, also showed a high diagnostic accuracy comparable to that of the insulin tolerance test [39]. The significant association between GH secretion and serum hs-CRP levels shown in our study should be confirmed in further studies using macimorelin to assess GH secretion.

In conclusion, this retrospective study revealed a significant and inverse association between GH secretion and serum hs-CRP levels, independent of atherosclerotic risk factors; metabolic complications of GHD, including obesity and fatty liver; and other anterior pituitary hormone deficiencies in patients with NF-PitNET and RCC. Postoperative changes in serum hs-CRP levels were significantly and inversely correlated with those in the peak GH response to GHRP-2. These results suggest an important protective role of GH secretion in increasing cardiovascular disease risk through inflammation, independent of the metabolic complications of GHD.

## Data Availability

Some or all datasets generated during and/or analyzed during the current study are not publicly available but are available from the corresponding author on reasonable request.

## Abbreviations

ALT: alanine aminotransferase
AST: aspartate aminotransferase
BMI: body mass index
CRP: C-reactive protein
GFR: glomerular filtration rate
GGT: γ-glutamyl transpeptidase
GHD: GH deficiency
GHRP: GH-releasing peptide
hs-CRP: high-sensitivity C-reactive protein
HbA1c: hemoglobin A1c
HDL: high-density lipoprotein
LDL: low-density lipoprotein
NF-PitNET: nonfunctioning pituitary neuroendocrine tumor
RCC: Rathke cleft cyst

## References

[1] Ho KK, 2007 GH Deficiency Consensus Workshop Participants. Consensus guidelines for the diagnosis and treatment of adults with GH deficiency II: a statement of the GH research society in association with the European society for pediatric endocrinology, lawson wilkins society, European society of endocrinology, Japan endocrine society, and endocrine society of Australia. Eur J Endocrinol. 2007;157(6):695‐700. 18057375 10.1530/EJE-07-0631

[2] Melmed S . Pathogenesis and diagnosis of growth hormone deficiency in adults. N Engl J Med. 2019;380(26):2551‐2562. 31242363 10.1056/NEJMra1817346

[3] Nishizawa H, Iguchi G, Murawaki A, et al Nonalcoholic fatty liver disease in adult hypopituitary patients with GH deficiency and the impact of GH replacement therapy. Eur J Endocrinol. 2012;167(1):67‐74. 22535644 10.1530/EJE-12-0252

[4] Yuen KCJ, Biller BMK, Radovick S, et al American Association of clinical endocrinologists and American college of endocrinology guidelines for management of growth hormone deficiency in adults and patients transitioning from pediatric to adult care. Endocr Pract. 2019;25(11):1191‐1232. 31760824 10.4158/GL-2019-0405

[5] Stochholm K, Gravholt CH, Laursen T, et al Incidence of GH deficiency—a nationwide study. Eur J Endocrinol. 2006;155(1):61‐71. 16793951 10.1530/eje.1.02191

[6] Rosén T, Bengtsson BÅ. Premature mortality due to cardiovascular disease in hypopituitarism. Lancet. 1990;336(8710):285‐288. 1973979 10.1016/0140-6736(90)91812-o

[7] Svensson J, Bengtsson BA, Rosen T, Oden A, Johannsson G. Malignant disease and cardiovascular morbidity in hypopituitary adults with or without growth hormone replacement therapy. J Clin Endocrinol Metab. 2004;89(7):3306‐3312. 15240607 10.1210/jc.2003-031601

[8] Bäck M, Yurdagul A Jr, Tabas I, Öörni K, Kovanen PT. Inflammation and its resolution in atherosclerosis: mediators and therapeutic opportunities. Nat Rev Cardiol. 2019;16(7):389‐406. 30846875 10.1038/s41569-019-0169-2 PMC6727648

[9] Koenig W . High-sensitivity C-reactive protein and atherosclerotic disease: from improved risk prediction to risk-guided therapy. Int J Cardiol. 2013;168(6):5126‐5134. 23978367 10.1016/j.ijcard.2013.07.113

[10] Kim-Mitsuyama S, Soejima H, Yasuda O, et al Reduction in hsCRP levels is associated with decreased incidence of cardiovascular events in Japanese hypertensive women but not in men. Sci Rep. 2020;10(1):17040. 33046765 10.1038/s41598-020-73905-4 PMC7550334

[11] Sesmilo G, Miller KK, Hayden D, Klibanski A. Inflammatory cardiovascular risk markers in women with hypopituitarism. J Clin Endocrinol Metab. 2001;86(12):5774‐5781. 11739438 10.1210/jcem.86.12.8087

[12] Deepak D, Daousi C, Javadpour M, et al The influence of growth hormone replacement on peripheral inflammatory and cardiovascular risk markers in adults with severe growth hormone deficiency. Growth Horm IGF Res. 2010;20(3):220‐225. 20185347 10.1016/j.ghir.2010.02.002

[13] McCallum RW, Sainsbury CA, Spiers A, et al Growth hormone replacement reduces C-reactive protein and large-artery stiffness but does not alter endothelial function in patients with adult growth hormone deficiency. Clin Endocrinol (Oxf). 2005;62(4):473‐479. 15807879 10.1111/j.1365-2265.2005.02245.x

[14] von Elm E, Altman DG, Egger M, et al Strengthening the reporting of observational studies in epidemiology (STROBE) statement: guidelines for reporting observational studies. BMJ. 2007;335(7624):806‐808. 17947786 10.1136/bmj.39335.541782.AD PMC2034723

[15] Chihara K, Shimatsu A, Hizuka N, et al A simple diagnostic test using GH-releasing peptide-2 in adult GH deficiency. Eur J Endocrinol. 2007;157(1):19‐27. 17609397 10.1530/EJE-07-0066

[16] Molitch ME, Clemmons DR, Malozowski S, Merriam GR, Vance ML, Endocrine Society. Evaluation and treatment of adult growth hormone deficiency: an endocrine society clinical practice guideline. J Clin Endocrinol Metab. 2011;96(6):1587‐1609. 21602453 10.1210/jc.2011-0179

[17] Fukuda I, Hizuka N, Muraoka T, Ichihara A. Adult growth hormone deficiency: current concepts. Neurol Med Chir (Tokyo). 2014;54(8):599‐605. 10.2176/nmc.ra.2014-0088 PMC4533495 25070016

[18] Kinoshita Y, Tominaga A, Usui S, et al The arginine and GHRP-2 tests as alternatives to the insulin tolerance test for the diagnosis of adult GH deficiency in Japanese patients: a comparison. Endocr J. 2013;60(1):97‐105. 23079545 10.1507/endocrj.ej12-0230

[19] Matsuo S, Imai E, Horio M, et al Collaborators developing the Japanese equation for estimated GFR. Revised equations for estimated GFR from serum creatinine in Japan. Am J Kidney Dis. 2009;53(6):982‐992. 19339088 10.1053/j.ajkd.2008.12.034

[20] Isojima T, Shimatsu A, Yokoya S, et al Standardized centile curves and reference intervals of serum insulin-like growth factor-I (IGF-I) levels in a normal Japanese population using the LMS method. Eur J Endocrinol. 2012;59(9):771‐780. 10.1507/endocrj.ej12-0110 22673406

[21] Sarlis NJ, Gourgiotis L, Koch CA, et al MR Imaging features of thyrotropin-secreting pituitary adenomas at initial presentation. AJR Am J Roentgenol. 2003;181(2):577‐582. 12876051 10.2214/ajr.181.2.1810577

[22] Miller M, Zhan M, Havas S. High attributable risk of elevated C-reactive protein level to conventional coronary heart disease risk factors: the third national health and nutrition examination survey. Arch Intern Med. 2005;165(18):2063‐2068. 16216995 10.1001/archinte.165.18.2063

[23] Stuveling EM, Hillege HL, Bakker SJ, Gans RO, De Jong PE, De Zeeuw D. C-reactive protein is associated with renal function abnormalities in a non-diabetic population. Kidney Int. 2003;63(2):654‐661. 12631131 10.1046/j.1523-1755.2003.00762.x

[24] Papanicolaou DA . Cytokines and adrenal insufficiency. Curr Opin Endocrinol Diabetes Obes. 1997;4(3):194‐198.

[25] Nagasaki T, Inaba M, Shirakawa K, et al Increased levels of C-reactive protein in hypothyroid patients and its correlation with arterial stiffness in the common carotid artery. Biomed Pharmacother. 2007;61(2-3):167‐172. 17383146 10.1016/j.biopha.2006.10.008

[26] Dandona P, Dhindsa S. Update: hypogonadotropic hypogonadism in type 2 diabetes and obesity. J Clin Endocrinol Metab. 2011;96(9):2643‐2651. 21896895 10.1210/jc.2010-2724 PMC3167667

[27] Osmancevic A, Ottarsdottir K, Hellgren M, Lindblad U, Daka B. High C-reactive protein is associated with increased risk of biochemical hypogonadism: a population-based cohort study. Endocr Connect. 2022;11(7):e220141. 35904226 10.1530/EC-22-0141 PMC9254292

[28] Sukhanov S, Higashi Y, Shai SY, et al IGF-1 reduces inflammatory responses, suppresses oxidative stress, and decreases atherosclerosis progression in ApoE-deficient mice. Arterioscler Thromb Vasc Biol. 2007;27(12):2684‐2690. 17916769 10.1161/ATVBAHA.107.156257

[29] Ratku B, Sebestyén V, Erdei A, Nagy EV, Szabó Z, Somodi S. Effects of adult growth hormone deficiency and replacement therapy on the cardiometabolic risk profile. Pituitary. 2022;25(2):211‐228. 35106704 10.1007/s11102-022-01207-1 PMC8894188

[30] Paepegaey AC, Genser L, Bouillot JL, Oppert JM, Clément K, Poitou C. High levels of CRP in morbid obesity: the central role of adipose tissue and lessons for clinical practice before and after bariatric surgery. Surg Obes Relat Dis. 2015;11(1):148‐154. 25393045 10.1016/j.soard.2014.06.010

[31] Ndumele CE, Nasir K, Conceiçao RD, Carvalho JA, Blumenthal RS, Santos RD. Hepatic steatosis, obesity, and the metabolic syndrome are independently and additively associated with increased systemic inflammation. Arterioscler Thromb Vasc Biol. 2011;31(8):1927‐1932. 21546603 10.1161/ATVBAHA.111.228262 PMC3148106

[32] Higashi Y, Sukhanov S, Shai SY, et al Insulin-like growth factor-1 receptor deficiency in macrophages accelerates atherosclerosis and induces an unstable plaque phenotype in apolipoprotein E-deficient mice. Circulation. 2016;133(23):2263‐2278. 27154724 10.1161/CIRCULATIONAHA.116.021805 PMC4899151

[33] Melmed S . Growth hormone. In: Bichet BG (ed.), The Pituitary. 5th ed. Elsevier; 2022:91‐129.

[34] Jørgensen JOL, Johannsson G, Barkan A. Should patients with adult GH deficiency receive GH replacement? Eur J Endocrinol. 2021;186(1):D1‐D15. 34714773 10.1530/EJE-21-0534

[35] Martel-Duguech LM, Jorgensen JOL, Korbonits M, et al ESE Audit on management of adult growth hormone deficiency in clinical practice. Eur J Endocrinol. 2020;184(2):321‐332. 10.1530/EJE-20-1180 33320830

[36] Sproston NR, Ashworth JJ. Role of C-reactive protein at sites of inflammation and infection. Front Immunol. 2018;9:754. 29706967 10.3389/fimmu.2018.00754 PMC5908901

[37] Szalecki M, Malinowska A, Prokop-Piotrkowska M, Janas R. Interactions between the growth hormone and cytokines—a review. Adv Med Sci. 2018;63(2):285‐289. 29579637 10.1016/j.advms.2018.03.001

[38] Ishida J, Saitoh M, Ebner N, Springer J, Anker SD, Haehling S. Growth hormone secretagogues: history, mechanism of action, and clinical development. JCSM Rapid Commun. 2020;3(1):25‐37.

[39] Garcia JM, Biller BMK, Korbonits M, et al Macimorelin as a diagnostic test for adult GH deficiency. J Clin Endocrinol Metab. 2018;103(8):3083‐3093. 29860473 10.1210/jc.2018-00665

